# Nucleating and Plasticization Effects in Drawn Poly(Lactic Acid) Fiber during Accelerated Weathering Degradation

**DOI:** 10.3390/polym10040365

**Published:** 2018-03-25

**Authors:** Masakazu Nishida, Tomoko Tanaka, Toshiyuki Tanaka, Yoshio Hayakawa

**Affiliations:** 1 National Institute of Advanced Industrial Science and Technology (AIST), 2266-98 Shimoshidami, Moriyama-ku, Nagoya 463-8560, Japan; tomo.tanaka@aist.go.jp (T.T.); hayakawa-y@aist.go.jp (Y.H.); 2 Owari Textile Research Center, Aichi Center for Industry and Science Technology, 35 Miyaura, Mabiki, Yamato-cho, Ichinomiya, Aichi 491-0931, Japan; toshiyuki_tanaka@pref.aichi.lg.jp

**Keywords:** polyesters, clay, drawing, degradation, mechanical properties, NMR, morphology

## Abstract

Changes in the polymer properties of poly(lactic acid) (PLA) fibers during drawing and degradation processes were analyzed using solid-state NMR, with the goal of elucidating morphological changes that influence fiber tensile properties. Combination of X-ray diffraction (XRD) and differential scanning calorimeter (DSC) indicated that the drawn PLA fibers consisted of different proportions of α crystalline and amorphous forms. ^13^C CP-MAS NMR spectra showed amorphous-like broad singlet signals, of which the full width at half maximum (FWHM) decreased with increasing crystallinity and crystal orientation. The *T*_1_H value decreased by interaction with additives and increased with increasing crystal orientation. The interaction with additives also reduced *T*_1_C values, which increased with increasing crystallinity. Use of organic clay enhanced the crystallization of high draw-ratio PLA fibers due to nucleation, which increased tensile strength; this effect gradually decreased with time during accelerated weathering. In contrast, the plasticization due to the addition of flexible polymers increased fiber elongation, which rapidly dropped during the degradation. Changes of FWHM, *T*_1_H, and *T*_1_C values indicated that the degradation occurred at sites within the amorphous portions of the PLA fibers containing organic clay, while the flexible polymers were preferentially degraded if they were present in the PLA fibers.

## 1. Introduction

In order to put the post-petroleum society into practice, greater development of biomass-based materials is required. Poly(lactic acid) (PLA) is presently the most researched biomass-based material; in-depth articles have been published concerning its manufacturing methods, material properties, and degradation [1]. Although PLA has the advantages of lower environmental impact, biocompatibility, and high-workability, it has its limitations, namely poor toughness, slow degradation rate, hydrophobicity, and lack of chemically reactive side-chains. To overcome these limitations, prior studies have investigated bulk and surface modifications [2] and improvement of mechanical and chemical processes [3]. In PLA manufacturing, crystallization is the most significant property that determines product characteristics [4], and previous studies have examined polymorphous crystallization and melting behavior using differential scanning calorimetry (DSC) and wide-angle X-ray diffraction (WAXD) [5,6]. To improve the mechanical properties of PLA enough for commercial applications, PLA-based nanocomposites were investigated to gain insights on controlling crystallinity and improving reinforcement [7]. 

Natural and man-made cellulose [8] and natural fibers [9] are typically used as reinforcement materials for PLA composites. As organic-modified clays are known to act as nucleating agents [4], PLA/clay composites have also been studied to evaluate their thermal and mechanical properties, biodegradation behavior [10], and the effects of modification and clay content on morphological and thermomechanical characterization [11]. Meanwhile, plasticization of PLA has been extensively studied to improve toughening and extend PLA’s application because plasticization enhances chain mobility from the amorphous phase onto the crystalline surface [4]. While poly(ethylene glycol) (PEG) is the most investigated plasticizer, blending of PLA with other flexible biodegradable polymers has also been explored to improve the toughness and the degradation rate. In one example, thermal and mechanical properties were studied for PLA blends with poly(ε-caprolactone) (PCL) [12]. In a second study of flexible biodegradable polymers, the impact strength of PLA was observed to increase when blended with poly(butylene succinate) (PBS) [13]. 

Owing to the characteristics outlined above, PLA manufacturing is most conveniently done using melt processing, i.e., extrusion, molding, thermoforming, and fiber spinning [3]. Melt spinning in particular has been actively studied for commodity and medical fiber applications because this method has been shown to effectively change the tensile strength, modulus, and morphology of PLA fibers [14]. A previous study examined several grades of PLA fiber obtained by high-speed melt spinning to characterize polymer crystallinity, orientation, and textile physical properties [15]. A two-step melt-spinning method has also been used to produce PLA fibers, which were studied for thermal and tensile properties and fiber morphology [16,17]. Past work has also examined the effects of crystallinity and fiber diameter on the mechanical and moisture absorption properties of melt-drawn PLA fibers [18]. Thermal analysis of PLA monofilaments using modulated DSC showed that hot fiber drawing increased the glass transition temperature (*T*_g_) and decreased heat capacity [19]. For textile applications, PLA/clay nanocomposite have also been produced to examine clay dispersion as well as thermal, mechanical, and shrinkage properties [20]. In samples of PLA/PBS fiber blends produced with a twin screw extruder and subsequent melt spinning, it was found that the inclusion of PBS decreased crystallinity and enhanced elasticity [21]. 

Controlling the degradation rate of PLA fiber is also a subject important to wider PLA utilization in many applications, and several studies on PLA fiber degradation have been published. The properties of PLA fibers made by melt spinning and drawing and then degraded by soil burial, compositing, and hydrolysis have been studied [22]. Near-infrared hyperspectral imaging showed that accelerated weathering predominately degraded the amorphous structure of the PLA [23]. To our knowledge, however, the effects of nucleation and plasticization on PLA fiber degradation have not been studied yet, especially at the molecular and nanometer scales.

Among instrumental analytical methods for biodegradable polymers, solid-state NMR has provided useful information not only on structure, but also molecular dynamics. To analyze nanoscale structure, the crystallinity and morphology of PLA have been investigated using ^13^C cross-polarization/magic angle spinning (CP-MAS) NMR [24,25]. For detailed structural analysis of PLA, the helical jump motions of α crystalline PLA have been studied using 2D ^13^C exchange NMR [26] as well as NMR crystallography of the α crystalline form, which was achieved using the valence and torsion angles calculated by ^13^C σ shielding [27]. The crystalline structural organization of solution-crystallized poly(l-lactic acid) (PLLA) was confirmed by instrumental analyses including ^13^C CP-MAS NMR [28]. Solid-state NMR is also useful for analyzing the degradation process of PLA; ^13^C CP-MAS NMR showed that amorphous PLA yielded the α crystalline form during hydrolytic degradation, while *T*_1__ρ_ analysis indicated that an increased regularity of the crystalline structure occurred at the same time [29]. The effects of hydrolytic degradation, crystallinity, and water absorption have all been examined using ^13^C CP-MAS NMR and *T*_1_C analyses [30]. Meanwhile, we have been studying application of solid-state NMR to biomass-based polymers in terms of industrial quality control. For example, we have examined the effects of manufacturing processes on PLA composites using a reactive extrusion machine and nuclear magnetic relaxation time analysis; to date, we have investigated PLA nanocomposites with organic montmorillonites [31] and PLA/poly(ε-caprolactone) (PCL) random copolymers [32]. Furthermore, we have also studied the molecular dynamics of biomass constituents in woody and herbaceous materials interacting with water molecules, using solid-state NMR spectra and nuclear magnetic relaxation times [33,34,35]. 

In the present work, we focused on PLA fiber made by melt-spinning in order to further demonstrate the application of solid-state NMR to a product form. As a first step, we examine the crystallinity and orientation of crystalline of the PLA fibers during the drawing by observing changes in ^13^C CP-MAS NMR spectra and nuclear magnetic relaxation times, along with observations using the XRD and DSC. According to the correlation between the polymer properties and the relaxation time, we studied the effects of nucleation and plasticization additives on PLA degradation processes. The difference between nucleating and plasticization additives at the molecular to nanometer levels, which is a significant factor for the manufacturing quality control, will reveal how PLA morphology influences the fiber tensile properties.

## 2. Materials and Methods 

### 2.1. Materials

Fiber grade poly(lactic acid) (PLA) pellet was purchased from Toyo Jyushi Co. Ltd. (Komaki, Japan). An organic-modified bentonite, S-Ben W, was used as a clay additive and was purchased from Hojun Co. Ltd. (Annaka, Japan). Poly(ε-caprolactone) (PCL) pellet was purchased from Daicel Chemical Industries Co. Ltd. (Osaka, Japan). Poly(butylene succinate) (PBS) was purchase from Showa Highpolymer Co. Ltd. (Tokyo, Japan). All materials were used without further purification.

### 2.2. Melt Spinning and Drawing of PLA Compounds

Melt spinning and drawing of PLA with the additives (organic clay and flexible polymers) as well as pure PLA were performed using a previously described procedure [22], which is summarized here. PLA and the additives were dried under vacuum at 80 °C for at least 3 h to remove moisture. The dried PLA and additives were placed into a Labo-plastomill 4C150 (Toyo Seiki Seisaku-sho Ltd., Tokyo, Japan) and then melt mixed at 190 °C and 55 rpm with a biaxial extruder to provide PLA compounds. The produced PLA compounds were designated as follows: (i) PLA (PLA without additives); (ii) 5% clay/PLA (PLA with 5% S-ben W); (iii) 10% clay/PLA (PLA with 10% S-ben W); (iv) 5% PCL/PLA (PLA with 5% PCL); and (v) 5% PBS/PLA (PLA with 5% PBS). Fibers of the PLA compounds were formed using a melt spinning machine. The PLA fibers were extruded at 200 °C and then drawn through boiled water (98 °C) using a combination of rollers at different rotational speeds. A draw ratio is defined as the rotational speed ratio between first and last rollers. Fibers of PLA compounds were manufactured using draw ratios of 0 (i.e., as melt-spun), 3, 4, and 6, and are labeled in this paper as 0 DR, 3 DR, 4 DR, and 6 DR, respectively.

### 2.3. Accelerated Weathering Degradation

The drawn PLA fibers were cut into 500 mm length and then formed into bundles of 15 pieces for accelerated weathering tests. The bundles were placed in a UV chamber of a Sunshine Weather Meter S80 (Suga Test Instruments Co. Ltd., Tokyo, Japan). The bundles were irradiated with a UV lamp at 65 °C and 50% relative humidity. The accelerated weathering tests were carried out for five different durations (200, 400, 600, 800, and 1000 h) for each PLA compound.

### 2.4. Fiber Chractrization

The X-ray diffraction (XRD) under Cu K*α* irradiation was measured on a RINT-Ultima II diffratometer (Rigaku Co. Ltd., Tokyo, Japan) operating at 40 kV and 40 mA. The fiber sample was cut to 18 mm lengths, which were aligned in one direction on a sample holder without overlapping. The diffractogram was recorded at 2θ angles between 5° and 40°. Differential scanning calorimeter (DSC) measurements were performed using a DSC-60 instrument (Shimadzu Co. Ltd., Kyoto, Japan). The sample (35 mg) was sealed in an aluminum pan and then heated from 30 to 250 °C at a heating rate of 10 °C/min under 250 mL/min N_2_ flow. An empty aluminum pan was used as a reference. Transmission electron microscopy (TEM) images were recorded using a JEOL JEM2100 (JEOL Ltd., Akishima, Japan) at an acceleration voltage of 200 kV. Ultrathin sections of 80–90 nm thick were prepared in epoxy resin by an ULTTOTOME (LKB, Bromma, Sweden) with an Ultra 3 mm, 45°, diamond knives (DIATOME Ltd., Nidau, Switzerland).

### 2.5. Solid-State NMR

The ^13^C cross-polarization/magic angle spinning (CP-MAS) NMR spectra were measured on a Varian 400 NMR system spectrometer with a Varian 4mm double-resonance T3 solid probe (Varian Inc., Palo Alto, CA, USA) at 100.56 MHz for ^13^C nuclei. Cut samples were placed in zirconia rotors of 4 mm in diameter and spun at magic angle at 15 kHz over a temperature range 20 to 24 °C. The CP-MAS was performed using a ramped-amplitude pulse at 2 ms contact time with a 2.5 μs π/2 pulse for the ^1^H nuclei. The spectra were collected with 40 ms acquisition periods over a 30.7 kHz spectral width in 1024 transients, with 5 s recycling time between the π/2 pulses. The relaxation time analyses were performed under the same acquisition conditions as the ^13^C CP-MAS NMR described above. The ^1^H spin-lattice relaxation time in the laboratory frame (*T*_1_H) was indirectly measured via detection of ^13^C resonance enhanced by cross-polarization, applied after a π pulse to ^1^H nuclei with the inversion recovery method. The ^13^C spin-lattice relaxation time in the laboratory frame (*T*_1_C) was measured with the conventional Torchia’s pulse sequence [36].

### 2.6. Tensile Testing

Tensile properties of the PLA fibers before and after accelerated weathering were measured using a Tensilon RTG 1310 (A&D Co. Ltd., Tokyo, Japan) at 20 °C and relative humidity 65%, where the cross head speed was set at 200 mm/min and the length of specimens was 200 mm. The tensile properties reported below represent average values of 10 trials for specimens before weathering degradation or 5 trials for specimens after weathering degradation, in accordance with the JIS L 1013 testing methods [37] for man-made filament yarn.

## 3. Results and Discussion

### 3.1. Fiber Chatactrization

Transparent fibers of each PLA compound were produced by spinning each composition at 190 °C. While 3 DR and 4 DR fibers were drawn in one step, the 6 DR fibers (except the PLA fiber blended with 5 wt % PCL) were drawn in two steps in order to prevent whitening. Thus, all fibers maintained their transparency after drawing. Furthermore, even after long periods of UV irradiation at 65 °C and 50% relative humidity, the degraded PLA fibers did not whiten but stayed transparent, except for very slight amber coloring of the PLA/clay fibers. 

**Figure 1:**
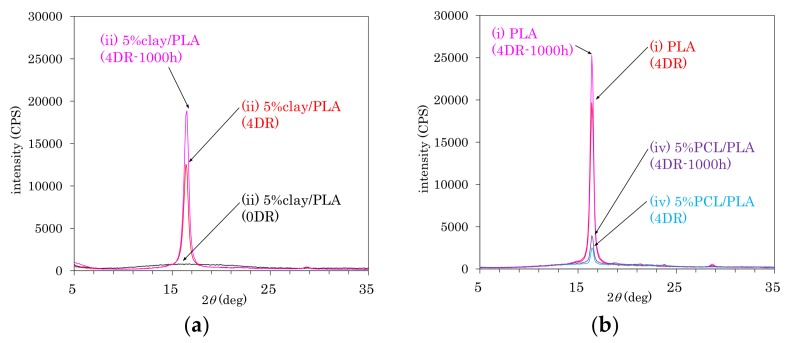
XRD diffraction patterns: (**a**) (ii) 5% clay/PLA; (**b**) (i) PLA and (iv) 5% PCL/PLA.

As shown in Figure 1, the crystal structures of PLA in fiber form were examined using X-ray diffraction (XRD). Figure 1a shows changes in the XDR patterns of 5% clay/PLA fiber (ii) due to the drawing and degradation. The as-formed 5% clay/PLA (0 DR) fibers showed no crystal patterns, which changed to a sharp α-crystalline peak (16.4°) due to the drawing (4 DR); at the same time, neither α’ and β crystalline peaks were observed. The intensity of α-crystalline peak increased and no other peaks appeared after the degradation testing (4 DR, 1000 h). The α-crystalline peak was also observed for other drawn fibers before and after the degradation, as shown in Figure 1b. While the 4DR PLA fiber (i) showed a sharp α-crystalline peak, the 4 DR 5% PCL/PLA fiber (iv) had a smaller α-crystalline peak overlapped with a broad amorphous peak. However, the α-crystalline peaks of both 4 DR PLA fiber (i) and 4 DR 5% PCL/PLA fiber (iv) increased after the degradation.

**Figure 2:**
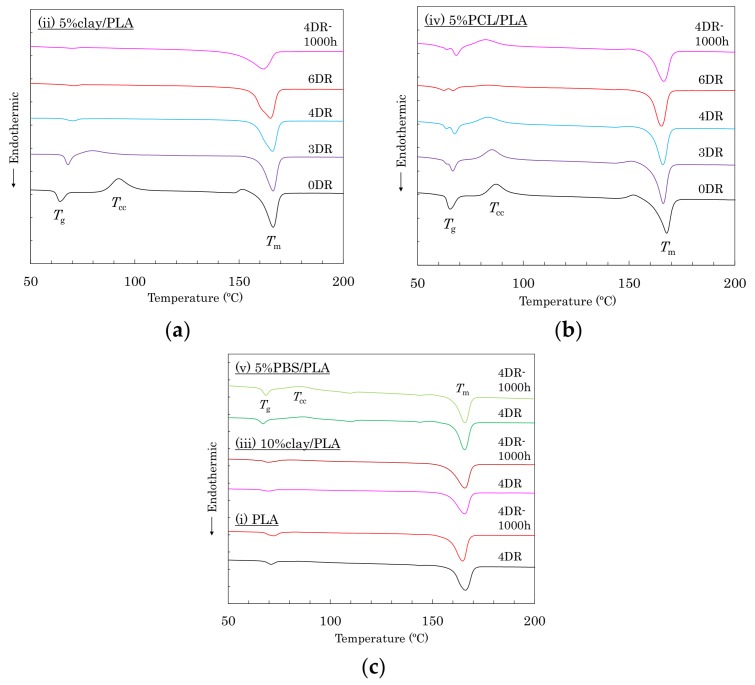
DSC heating curves: (**a**) (ii) 5% clay/PLA; (**b**) (iv) 5% PCL/PLA; (**c**) 4 DR and 1000 h degraded (i) PLA, (iii) 10% clay/PLA, and (v) 5% PBS/PLA.

As shown in Figure 2, the differential scanning calorimeter (DSC) analysis also indicated the effects of nucleating and plasticization additives for PLA crystallinity. The as-formed (0 DR) fibers showed similar DSC curves, which had glass transitions (*T*_g_) that overlapped with entropy relaxation, cold crystallization (*T*_cc_), and melting (*T*_m_) peaks. The *T*_g_ peak became smaller and shifted to higher temperature, whereas the *T*_cc_ peak also became smaller but shifted to lower temperature, depending on the draw ratio. These changes of *T*_g_ and *T*_cc_ mean that the drawing increased the crystallinity of PLA with increasing draw ratio. The peak reduction and shift rates of the 5% clay/PLA fiber (Figure 2a) were larger than those of the 5% PCL/PLA fibers (Figure 2b). According to the DSC results of other PLA fibers (Figure 2c), the organic clay enhanced the crystallization of PLA, while the presence of PCL suppressed PLA crystallization. Furthermore, the *T*_g_ peak decreased in the 4 DR 5% clay/PLA fiber; however, no changes of DSC curves appeared in other fibers after the degradation (4 DR-1000 h).

**Figure 3:**
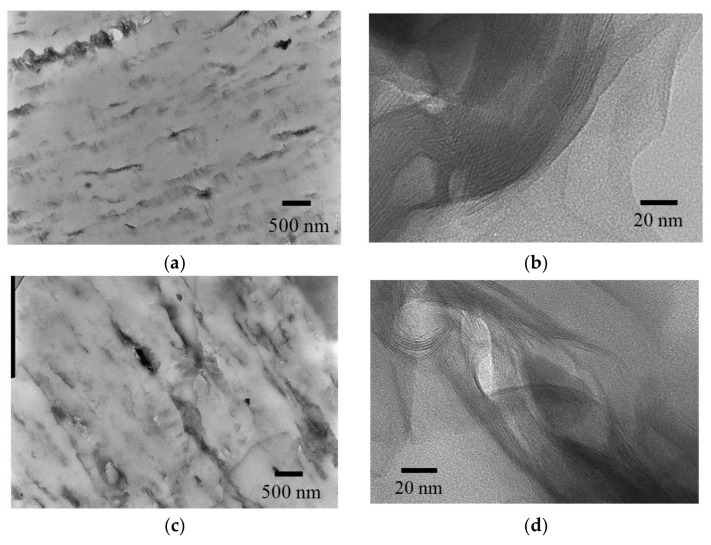
TEM micrographs: (**a**) (ii) 4 DR 5% clay/PLA (10k ×); (**b**) 4 DR 5% clay/PLA (200k ×); (**c**) 4 DR 5% clay/PLA 1000 h degradation (10k ×); (**d**) 4 DR 5% clay/PLA 1000 h degradation (200k ×).

For the PLA fiber containing organic clay, the dispersion of the clay particles is a significant factor controlling the fiber properties. Different affinity between layered silica and organophilic polymers caused three different nanostructures, that is, phase separated, intercalated, and exfoliated [7]. Our previous reports showed that organic clay existed in intercalated form within PLA clay composites produced not only by the biaxial extruder [31], but also by the reactive extruder [38]. To examine dispersion and nanostructure of the PLA fiber containing the organic clay, direct observation via transmission electron microscope (TEM) imaging was performed for 4 DR 5% clay/PLA (ii) before and after the accelerated weathering test, as shown in Figure 3. The image of the specimen before the degradation showed homogenously dispersed clays at low magnification (Figure 3a). These dispersed clays showed intercalated form while the exfoliation could not be observed at high magnification (Figure 3b). After the degradation, the organic clays still homogenously dispersed, but several cracks were also observed (Figure 3c). Even after the degradation, the intercalated form was the major clay morphology without the exfoliation; however, somewhat disordered layers could be observed. 

### 3.2. ^13^C CP-MAS NMR Spectra of PLA Fibers

**Figure 4:**
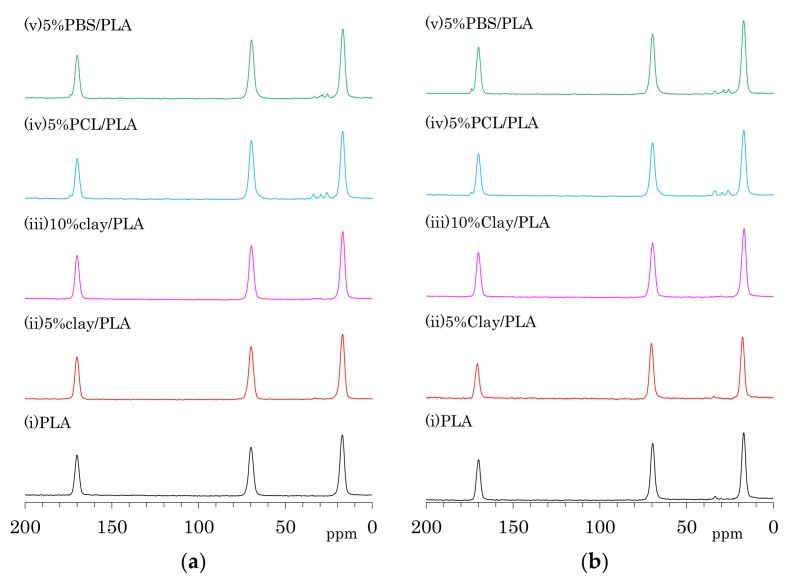
^13^C CP-MAS NMR spectra of PLA compounds: (**a**) before drawing; (**b**) after drawing at a draw ratio of 4.

The ^13^C CP-MAS NMR spectra of as-formed PLA fibers are shown in Figure 4a. One can see three symmetry singlet signals at 170 ppm (C=O), 70 ppm (CH), and 17 ppm (CH_3_) in the spectrum of PLA. These PLA signals were almost unchanged with the addition of organic clay as well the flexible polymers PCL and PBS. The PLA fiber with 5 wt % PCL addition (iv) showed additional peaks at 173 ppm (C=O), 65 ppm (CH_2_*C*H_2_O, overlapped with PLA), 33 ppm (CH_2_*C*H_2_C=O), 30 ppm (*C*H_2_CH_2_O), and 26 ppm (*C*H_2_*C*H_2_CH_2_C=O, overlapping two CH_2_ signals). Signals of PBS in 5%PBS/PLA (e) could be also assigned as follows: 174 ppm (C=O), 65 ppm (*C*H_2_C=O, overlapped with PLA), 28 ppm (CH_2_*C*H_2_O), and 25 ppm (*C*H_2_CH_2_O). 

The ^13^C CP-MAS NMR spectra of the PLA fibers made with a draw ratio of 4 (4 DR) are shown in Figure 4b. The 4 DR PLA fibers showed signal patterns that were similar to their respective PLA compositions before drawing. A previous report on the ^13^C CP-MAS NMR spectra of PLA showed that the α crystalline form had narrow and split signals, while the amorphous form showed broad singlet signals [24]. Furthermore, the α crystalline polymer made by solution crystallization also showed the narrow split signals in ^13^C CP-MAS NMR [28]. According to the ^13^C CP-MAS NMR spectral assignments in these previous reports, no PLA compositions exhibited an α crystalline signal; only a singlet amorphous-like signal appeared to be present both before and after fiber drawing. In our previous study of drawn PLA fiber, however, near-infrared (NID) hyperspectral imaging showed the existence of the crystalline form [23]. Another previous report showed the existence of the crystalline form in a similar PLA draw process, which was confirmed using X-ray diffraction (XRD) [22]. In the present study, the XRD results obviously showed α crystalline peak for both 4 DR PLA (i) and 5% clay/PLA fibers. Furthermore, our DSC results indicated that both 4 DR 5% clay/PLA (ii) and 5% PCL/PLA (iv) fibers had different crystallinity from the 0 DR fibers. By considering these previous reports about the drawn PLA fibers and combining them with our XRD and DSC results, the similar singlet amorphous-like signal for the different crystallinity was judged to be probably caused by a broadened α crystalline signal overlapped with the amorphous signal. 

**Figure 5:**
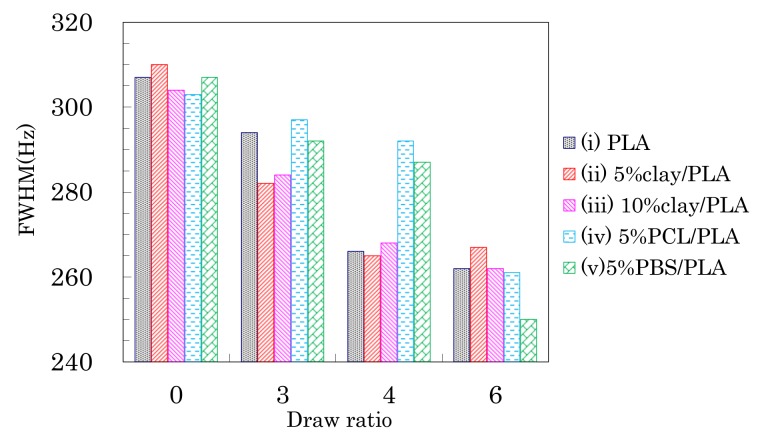
Full width at half maximum (FWHM) values of CH signal peaks of PLA fibers over a range of draw ratios.

Although the drawing of PLA compound fibers did not change the signal patterns of the ^13^C CP-MAS NMR spectra, the full width at half maximum (FWHM) of the PLA signals decreased depending on the fiber draw ratio as shown in Figure 5. All PLA fibers showed similar FWHM values before the drawing, even though the organic clay contained trace amounts of paramagnetic metal ions. Although the FWHM values of all PLA fibers decreased with increasing draw ratio, a marked FWHM decrease appeared at a different ratio for each PLA fiber. That is, the FWHM values of PLA fibers without additives (i) and with the organic clay (ii, iii) significantly decreased at 4 DR while the FWHM values of the PLA fibers with flexible polymers (iv, v) dramatically decreased at 6 DR. Previous studies have noted changes due to the drawing of PLA fibers, in particular an increase in crystallinity [16] and increases in orientation [39]. Changes to crystalline orientation appeared to depend on the draw ratio and are evident in Figure 5 as a regular decrease of the FWHM values. In contrast, the onset of crystallization appears as a significant decrease of the FWHM values, corresponding to the draw ratio at which the *T*_cc_ peak in the DSC analysis disappears. Therefore, the FWHM of the NMR signal for a PLA fiber can be an index not only for the crystallinity, but also for the orientation of the crystalline form.

### 3.3. Nuclear Magnetic Relaxation Times of PLA Fibers

**Figure 6:**
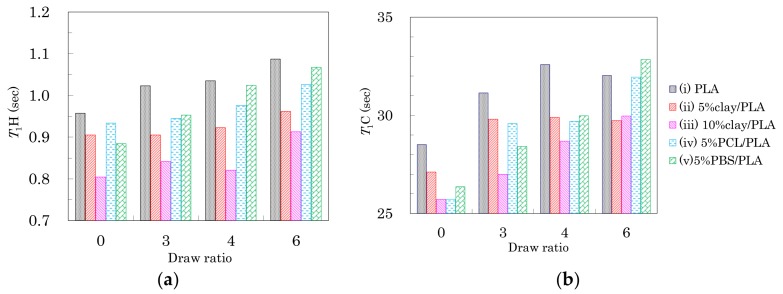
*T*_1_H values (**a**) and *T*_1_C values (**b**) of the CH signal from PLA fibers versus fiber draw ratio.

The changes in spin-lattice relaxation times in the laboratory frame (*T*_1_) of the PLA fibers are summarized in Figure 6. The *T*_1_ values for ^1^H nuclei (*T*_1_H) are shown in Figure 6a. Before the drawing (DR = 0), the addition of organic clay and flexible polymers caused the *T*_1_H values to decrease. Because the addition of clay and flexible polymers both increased the heterogeneity of the PLA fibers, the decease of the fiber *T*_1_H value was caused by the enhancement of spin-lattice relaxation due to the inhomogeneous portions of the fiber. The reduction in *T*_1_H values for the PLA clay fibers was proportional to the amount of clay added. 

After the drawing, the *T*_1_H values of the fibers increased depending on the draw rate. The increase of *T*_1_H value due to the drawing was obvious for the PLA fibers with/without the flexible polymers (i, iv, v). The orientation of the crystalline form affected the overall macroscopic structure of the polymer, resulting in a decrease in fiber heterogeneity. Consequently, the suppression of the spin-lattice relaxation due to greater inhomogeneity helped to maintain higher *T*_1_H values for the clay-free PLA fibers (i, iv, v). A smaller increase of *T*_1_H value was observed for the PLA fibers with the organic clay (ii, iii) because the relaxation via paramagnetic metal ions compensated for the increases of crystallinity and orientation. From the above results, the *T*_1_H value was not only decreased by the interaction with the nucleating and plasticization additives, but also increased with increasing orientation of the crystalline form. 

The *T*_1_ values for ^13^C nuclei (*T*_1_C) of the CH group in the PLA fibers are shown in Figure 6b. Before fiber drawing (DR = 0), the *T*_1_C values of the PLA modified with organic clay and flexible polymers were less than that of the unmodified PLA. These decreases in *T*_1_C values were caused by the enhancement of the spin-lattice relaxation due to the presence of inhomogeneity in the fiber, similar to the effect seen in *T*_1_H values. Moreover, the *T*_1_C values of the PLA fibers increased depending on the draw ratio; this increase of *T*_1_C values was caused by the crystallization as discussed in a previous report showing that PLA in its crystalline form had larger *T*_1_C values than the amorphous PLA [30]. Therefore, the increase in *T*_1_C values due to the drawing was caused not only by the suppression of the spin-lattice relaxation via the increasing inhomogeneity, but also by an increase in the crystalline nature of the PLA fibers. According to the results of DCS curves as well as the FWHM of the ^13^C CP-MAS NMR spectra, the greater increase of the *T*_1_C value corresponded to an initial step of crystallization. 

### 3.4. Tensile Properties of the Drawn PLA Fibers

**Figure 7:**
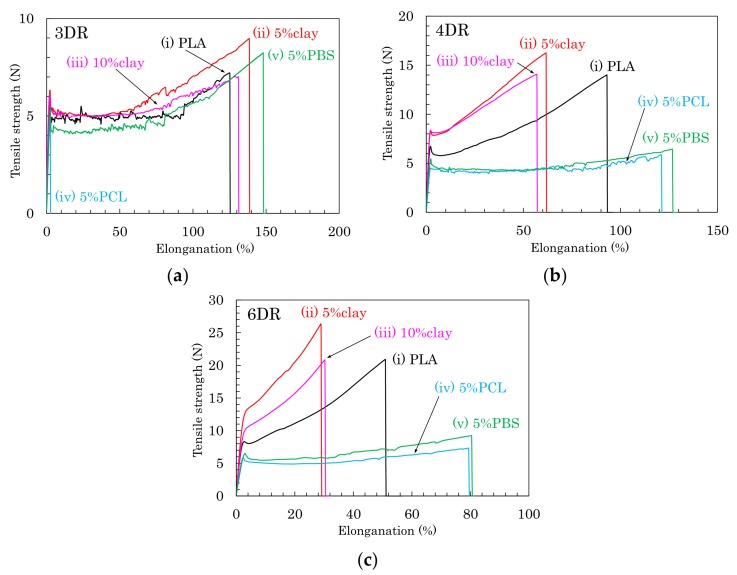
Stress-strain curves of PLA fibers formed using different draw ratios: (**a**) 3 DR; (**b**) 4 DR; (**c**) 6 DR.

**Figure 8:**
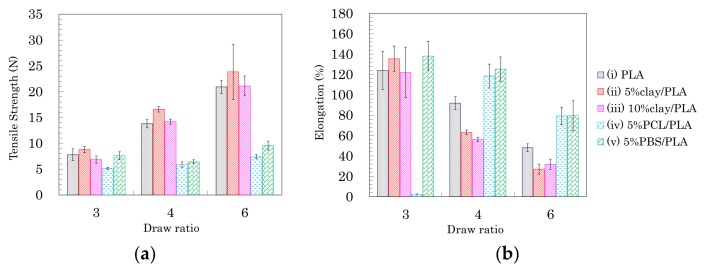
Tensile strength (**a**) and elongation (**b**) of PLA fibers formed using different draw ratios.

Based on the above results of the crystallinity and orientation of crystalline form, the effects of nucleating and plasticization additives were examined for tensile properties of the drawn PLA fibers. Figure 7 shows typical stress–strain curves of PLA compound fibers for each draw ratio. Figure 8 summarizes the tensile strengths and elongations of PLA compound fibers at several drawing ratios. Except for the 5% PCL/PLA (iv) samples, the PLA fibers formed at the low draw ratio (3 DR) showed no significant effect of the additives on tensile properties; nearly similar stress–strain curves were obtained for fibers with and without additives (Figure 7a). At higher draw ratios (4 DR, 6 DR), the crystallinity increased with increasing draw ratio, resulting in increased tensile strength in the pure PLA fiber (i) as well as the clay/PLA fibers (ii, iii). Especially in the clay/PLA fibers, the tensile strength increased at low elongation with reinforcement of the nucleating additive (Figure 7b,c).

Even at 6 DR, in the PLA fibers with flexible polymers (iv, v), the tensile strength did not increase even though those PLA fibers had higher crystallinity (Figure 7c). The tensile strength increased with the draw ratio for the PLA fibers (i–iii); however, the flexible polymers (iv, v) suppressed the increase of tensile strength due to the drawing (Figure 8a). Meanwhile, the elongation decreased with increasing draw ratio, regardless of crystallinity tendency (Figure 8b). As the draw ratio increased, the organic clay reduced the elongation greatly, with a lesser reduction observed for the blends with flexible polymers. In other words, the increase in tensile strength was affected not by the plasticization additive but by the nucleating additive, although the decrease of the elongation was affected by the presence of both additives. 

As shown in the earlier subsections, the fiber drawing increased the PLA crystallinity as well as the crystalline orientation. For the PLA fibers manufactured in the present study, the *T*_1_H change was dominated by the orientation of the crystalline form (Figure 6a), while the *T*_1_C change was mainly affected by the crystallization (Figure 6b). The tensile strength and elongation of the pure PLA fiber (i) and the clay/PLA fibers (ii, iii) changed gradually with the draw ratio, unlike the T_1_C changes, which reflected the change in crystallization. That is, the changes of tensile strength and elongation of these PLA fibers at the highest draw ratio (6 DR) were mainly caused by the crystalline orientation, while the elongation of the PLA fibers with flexible polymers (iv, v) markedly decreased at 6 DR. The elongation changes were mainly caused by the crystallization, because the elongation change with increasing draw ratio was similar to changes in the *T*_1_C value.

### 3.5. Accelerated Weathering Degradation—Changes of ^13^C CP-MAS NMR Spectra

**Figure 9:**
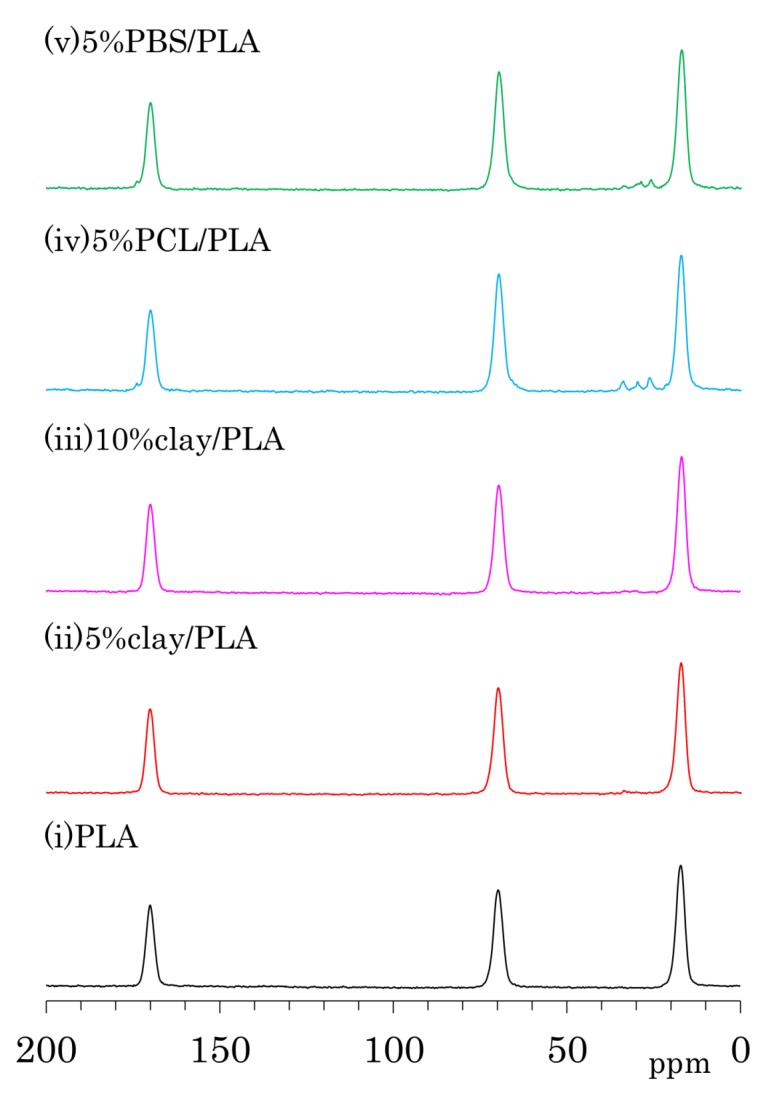
^13^C CP-MAS NMR spectra of 4 DR PLA fibers after 1000 h of accelerated weathering tests.

According to both XRD (Figure 1) and DSC (Figure 2) analyses, accelerated weathering degradation did not significantly change the crystallinity of the 4 DR PLA fibers. Meanwhile, the TEM analysis showed several cracks that were observed at low magnification, although the intercalated morphology appeared almost unchanged at high magnification. In order to examine changes due to degradation at molecular to nanometer scales, the ^13^C CP-MAS NMR spectrum of the 4 DR PLA fibers after 1000 h of weathering was measured as shown in Figure 9. The weathered 4 DR PLA fibers showed signal patterns similar to those of their unweathered state and to those of the PLA fibers as melt-spun (Figure 4). The hydrolytic degradation-produced singlet ^13^C CP-MAS NMR signal overlaps with the shouldered narrow and split signals of the *α* crystalline PLA [29]. In the degradation of all fibers, however, not only narrow and split signals, but also other terminal substituent signals could not be observed. Therefore, 4 DR PLA fibers after 1000 h of degradation maintained their crystallinity and crystalline form without α crystalline growth and release of monomers. 

**Figure 10:**
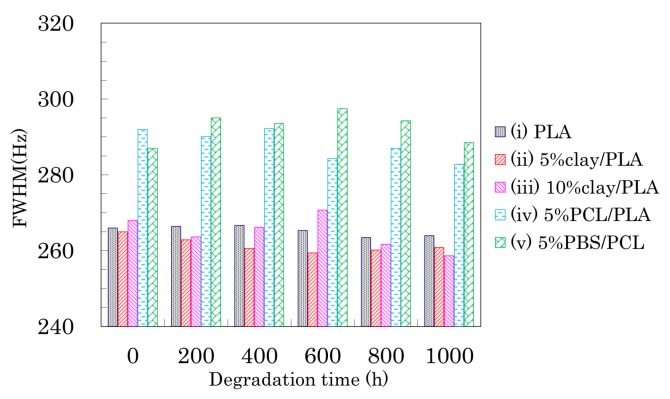
FWHM of CH signal from 4 DR PLA fibers plotted versus degradation time.

Changes in the FWHM of the CH signals due to weathering are shown in Figure 10. A monotonic decrease of the FWHM values due to the degradation was not observed, although the FWHM monotonically decreased with increasing draw ratio.

For the 4 DR PLA fiber without additives (i) and the 4 DR 5% clay/PLA fiber (ii), the FWHM values slightly decreased with increasing degradation time. For the other 4 DR PLA fibers (iii–v), the FWHM values appeared to scatter more, while also slightly decreasing with degradation time. The initial FWHM values showed obvious decreases with increasing draw ratio (Figure 5), suggesting that the fiber drawing enhanced crystallization as well as the orientation of the crystalline regions. During the degradation, the slight decrease of the FWHM value was caused by a slight increase in crystalline content because the weathering did not increase the orientation of the crystalline portions of the fibers. 

### 3.6. Accelerated Weathering Degradation—Changes of Nuclear Magnetic Relaxation Times

**Figure 11:**
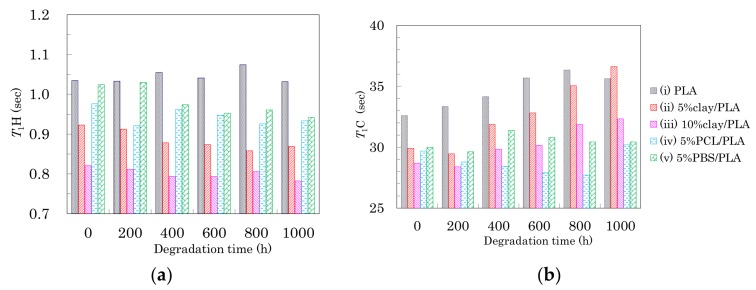
*T*_1_H values (**a**) and *T*_1_C values (**b**) of 4 DR PLA fibers plotted versus degradation time.

Changes in *T*_1_ of the PLA fibers with degradation time are summarized in Figure 11. In the drawn PLA fibers (Figure 6a), the *T*_1_H value was increased by the suppression the spin-lattice relaxation due to increasing inhomogeneity, which decreased not only as the interaction with the nucleating and plasticization additives increased, but also as the orientation of the crystalline regions increased. While the degradation of the 4 DR PLA fiber without additives (i) caused their *T*_1_H values to drift, the other 4 DR PLA fibers (ii–v) exhibited decreases, as shown in Figure 11a. The decrease of *T*_1_H values for the 4 DR PLA fibers indicated that the degradation did not increase the crystalline orientation, similar to the case with FWHM values. Therefore, the degradation caused an increase in the PLA heterogeneity, which increased with increasing interaction of the PLA fibers with the additives (ii–v), due to the degradation.

The *T*_1_C values of the CH group in the PLA fibers are plotted versus degradation time in Figure 11b. The *T*_1_C values of both 4 DR PLA fiber without additives (i) and 4 DR PLA clay fibers (ii, iii) roughly increased with degradation time. Meanwhile, the *T*_1_C values of both 4 DR PLA fibers with flexible polymers (iv, v) drifted throughout the degradation testing. In a previous study employing NIR imaging, we reported that the amorphous regions of the PLA fiber were decomposed by the same accelerated weathering degradation [23]. Considering the previous results using NIR imaging and the present results focusing on relaxation times, the accelerated weathering appears to have decomposed the amorphous portions of the polymers, resulting in an increase of crystallinity that suppresses the *T*_1_C relaxation. Furthermore, the decomposed amorphous material became defects, which increased inhomogeneity and enhanced the *T*_1_H relaxation. The suppression of the *T*_1_C relaxation due to decreases in the amorphous portions of the fibers was not effective for the 4 DR PLA blend fibers (iv, v), but did effect the 4 DR PLA fiber without additives (i) as well as the 4 DR fibers with clay (ii, iii) because of different crystalline contents.

### 3.7. Accelerated Weathering Degradation—Changes of Tensile Properties

**Figure 12:**
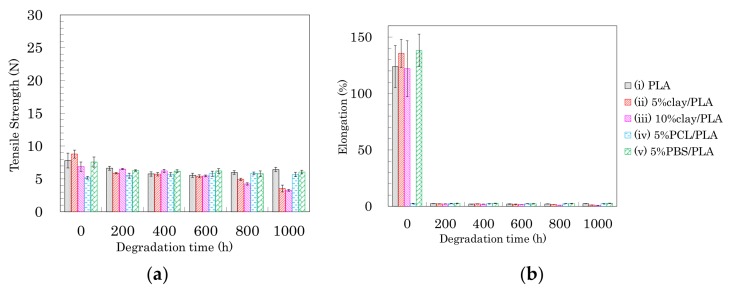
Tensile strength (**a**) and elongation (**b**) of 3 DR PLA fibers plotted versus degradation time.

**Figure 13:**
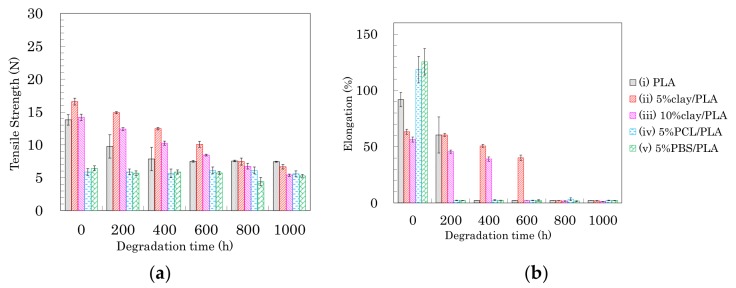
Tensile strength (**a**) and elongation (**b**) of 4 DR PLA fibers plotted versus degradation time.

**Figure 14:**
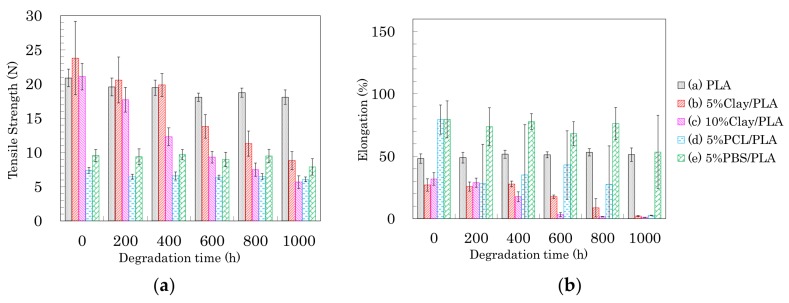
Tensile strength (**a**) and elongation (**b**) of 6 DR PLA fibers plotted versus degradation time.

The effects of accelerated weathering on the modified PLA fibers were examined using tensile tests and solid-state NMR analyses. All PLA fibers retained the capacity for tensile testing after the accelerated weathering. Changes of tensile strength and elongation due to the accelerated weathering are summarized in Figure 12, Figure 13 and Figure 14. The changes in tensile properties for 3 DR PLA fibers with increased weathering time are shown in Figure 12 (a: tensile strength, b: elongation).

Since 3 DR PLA fibers had lower crystallinity than those of fibers drawn at higher ratios, the tensile strengths were lower, so the decreases in tensile strength with degradation time of these fibers were small. The tensile strength of 3 DR PLA fiber (i) decreased after 400 h and then maintained a constant value to 1000 h. In the cases of the 3 DR PLA clay fibers (ii, iii), the tensile strength monotonically decreased over the full 1000 h test. The tensile strength of both 3 DR 5% PCL/PLA fiber (iv) and 3 DR 5% PBS/PLA fiber (v) remained nearly constant throughout the degradation testing. Fiber elongation changes with accelerated weathering were similar among all the fiber samples, even though the 3 DR PLA fibers had different initial elongation values. The elongations of all 3 DR PLA fibers promptly dropped to a few percent after only 200 h of weathering; that is, low crystallinity increased the degradation speed of the 3 DR PLA fibers.

The changes in tensile properties for 4 DR PLA fibers with degradation time are shown in Figure 13 (a: tensile strength, b: elongation). Each 4 DR PLA fiber exhibited conspicuous changes in tensile strength and elongation over the weathering tests because of increasing crystallinity. As was the case with the 3 DR fibers, the tensile strength of 4 DR PLA fiber (i) decreased until 400 h, while the decrease in tensile strength for the 4 DR PLA fibers (ii, iii) continued to 1000 h. Although both 4 DR 5%PCL/PLA fiber (iv) and 4 DR 5%PBS/PLA fiber (v) initially exhibited values of tensile strength that were lower than pure PLA, their tensile strengths scarcely decreased during weathering, even after 1000 h. The changes in elongations with degradation time were characteristic for each 4 DR PLA fiber, unlike those of the 3 DR PLA fibers. The weathering test time at which elongation of the 4 DR PLA fiber dropped to a few percent was in the following order: 5% PCL/PLA (iv) = 5% PBS/PBS (v) (200 h) < PLA (i) (400 h) < 10% clay/PLA (iii) (600 h) < 5% clay/PLA and (ii) (800 h). Judging by this elongation trend, the nucleation delayed degradation while the plasticization promoted degradation because of the different crystallinity of the 4 DR PLA fibers.

The changes of tensile properties during the weathering tests for 6 DR PLA fibers are shown in Figure 14 (a: tensile strength, b: elongation). The higher crystallinity and orientation of the crystalline form suppressed the degradation of the 6 DR PLA fiber (i), for which tensile strength and elongation only slightly decreased even after 1000 h of weathering. Although the organic clay also increased the crystallinity and the orientation of the 6 DR fibers, the nucleation promoted the degradation of the 6 DR fibers, contrary to the correlation observed in the 4 DR fibers. The 6 DR clay/PLA fibers showed some effect of clay content, as the 5% clay fibers (ii) degraded more slowly than the 10% clay fiber (iii), similar to the 4 DR clay/PLA fibers. Furthermore, the higher crystallinity and orientation of the crystalline form suppressed the degradation of the PLA fibers with the flexible polymers (iv, v); the decrease of tensile strength during the degradation was restrained. Although the elongation of 6 DR 5% PCL/PLA fiber (iv) rapidly decreased in the first 200 h of weathering, that of 6 DR 5% PBS/PLA fiber (v) remained at 50% until 800 h. This suggests that PBS in the 6 DR PLA fiber added greater stability against degradation, probably due to higher crystallinity, compared with other drawn PLA-polymer blend fibers.

### 3.8. Molpholiogical Changes Due to Accelerated Weathering Degradation

**Figure 15:**
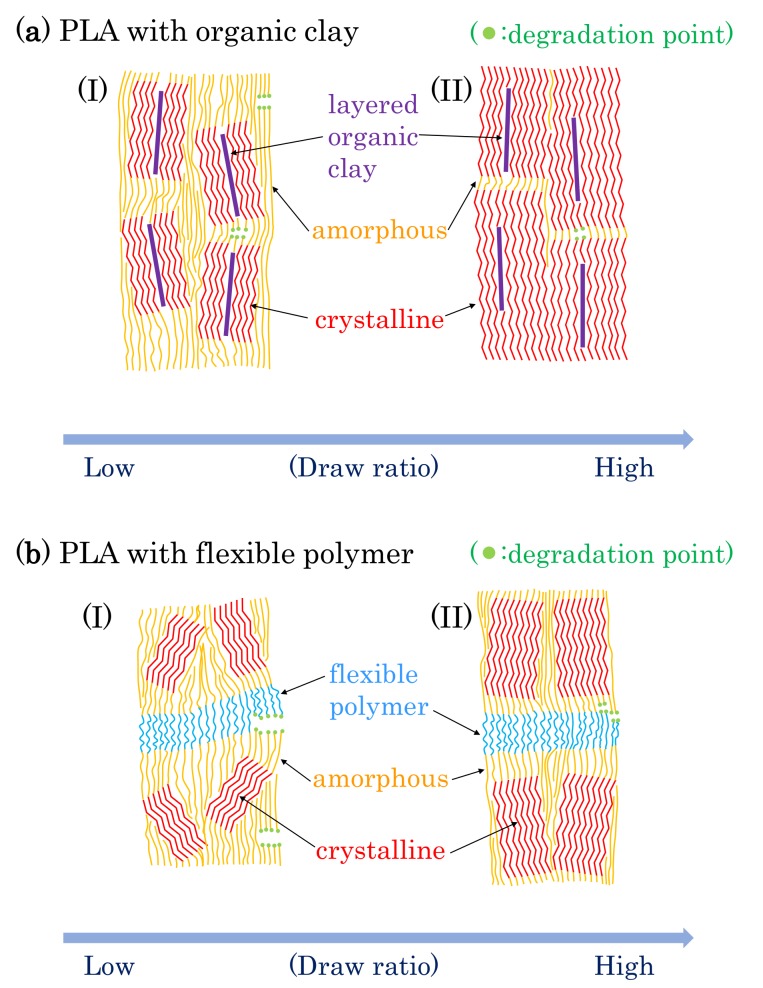
Proposed model of morphological changes in PLA fibers with drawing and degradation.

Based on the results described above, possible schemes of morphological changes within the PLA fibers are shown in Figure 15. Crystallization of PLA is predominant near the layered organic clay, which acts as a nucleation center. Before fiber drawing or at low fiber draw ratios, the crystalline regions are randomly placed and crystallinity is low (Figure 15(aI)). At higher fiber draw ratios, crystallinity increases and the crystalline becomes more highly oriented (Figure 15(aII)). Since the amorphous portions of the polymer are predominately decomposed by the accelerated weathering (see degradation point in Figure 15a), the crystallinity is further increased with the accelerated weathering; this view is supported by the increase of the *T*_1_C value. Since the layered clays disordered at the same time, the degradation evidently occurred near the boundary of the layered clay as shown in the TEM observation (Figure 3c), leading to a gradual decrease in the elongation for 4 DR and 6 DR clay/PLA fibers.

Flexible polymers, such as PCL and PBS, form a three-phase system with the PLA consisting of amorphous and crystalline phases. Before fiber drawing or for low draw ratios, the crystalline is randomly placed with low crystallinity for the PLA fibers in the presence of the flexible polymers, similar to the case with clay additives (Figure 15(aI)). At higher draw ratios, although the crystalline portions of the PLA are more oriented, the increase in crystallinity is lower than that in the PLA fibers due to the plasticization effect (Figure 15(aII)). Since the *T*_1_C value does not increase with increasing degradation time, the crystallinity of the PLA was unchanged by the accelerated weathering. This result indicates that the accelerated weathering attacks at sites within the flexible polymer (see degradation point in Figure 15b). Accordingly, the elongation rapidly decreases with weathering time because it is derived from the flexible polymers, insofar as the flexible polymer has resistance against the degradation, such as PBS in the 6 DR PLA fiber.

## 4. Conclusions

In order to extend application of solid-state NMR to support the manufacturing of biomass-based polymers, this study examined the effects of nucleating and plasticization additives on the fiber drawing and degradation processes of poly(lactic acid) fibers. The ^13^C CP-MAS NMR spectra showed broadened singlet signals, which are assigned to the α crystalline form overlapped with the amorphous signal, by considering the XRD and DSC results. The FWHM values of the NMR signal gradually decreased with increasing crystal orientation, while they step-wise decreased with increasing crystallinity. The *T*_1_H and *T*_1_C values decreased due to the interaction with the additives. At the same time, the *T*_1_H value increased with increasing crystal orientation, while the *T*_1_C value increased with increased crystallinity. These correlations between the polymer properties and the relaxation times applied to changes of fiber tensile properties observed after accelerated weathering degradation. Since lower draw ratio PLA fibers had lower crystallinity, their elongations rapidly dropped with degradation time, regardless of the presence of nucleating and plasticization additives. For the PLA fibers having higher crystallinity and a more highly oriented crystalline form, the nucleating additives reinforced the tensile strength, while they enhanced degradation near the boundaries of the layered clay. Even though the crystallinity and the orientation increased in the high draw ratio PLA-polymer blend fibers, the elongation rapidly deceased during degradation testing. The relaxation time analyses showed that the PLA crystallinity increased with the decomposition of amorphous PLA during the degradation of the PLA-clay fibers, while it was the flexible polymers that predominately decomposed during the degradation of the PLA-polymer blend fibers. Thus, the morphology of dispersed clay as well as the crystallinity of the flexible polymer should be considered to control the degradation of the PLA fibers. In the future, mechanical properties of other biomass-based materials will be investigated in terms of morphology, using combinational analyses of solid-state NMR spectra and relaxation times.
